# Natural Product Inspired Novel Indole based Chiral Scaffold Kills Human Malaria Parasites via Ionic Imbalance Mediated Cell Death

**DOI:** 10.1038/s41598-019-54339-z

**Published:** 2019-11-28

**Authors:** Poonam Dangi, Ravi Jain, Rajanikanth Mamidala, Vijeta Sharma, Shalini Agarwal, Chandramohan Bathula, M. Thirumalachary, Subhabrata Sen, Shailja Singh

**Affiliations:** 1 Department of Life Science, Shiv Nadar University, Gautam Buddha Nagar, 201314 India; 2 Jawaharlal Technological University, Kukatpally, 500072 Hyderabad India; 3 International Centre for Genetic Engineering and Biotechnology, New Delhi, 110067 India; 4 Department of Chemistry, Shiv Nadar University, Gautam Buddha Nagar, 201314 India; 5 Special Centre for Molecular Medicine, Jawaharlal Nehru University, New Delhi, 110067 India

**Keywords:** Parasite biology, Molecular medicine, Infection

## Abstract

Natural products offer an abundant source of diverse novel scaffolds that inspires development of next generation anti-malarials. With this vision, a library of scaffolds inspired by natural biologically active alkaloids was synthesized from chiral bicyclic lactams with steps/scaffold ratio of 1.7:1. On evaluation of library of scaffolds for their growth inhibitory effect against malaria parasite we found one scaffold with IC_50_ in low micro molar range. It inhibited parasite growth via disruption of Na^+^ homeostasis. P-type ATPase, PfATP4 is responsible for maintaining parasite Na^+^ homeostasis and is a good target for anti-malarials. Molecular docking with our scaffold showed that it fits well in the binding pocket of PfATP4. Moreover, inhibition of Na^+^-dependent ATPase activity by our potent scaffold suggests that it targets parasite by inhibiting PfATP4, leading to ionic imbalance. However how ionic imbalance attributes to parasite’s death is unclear. We show that ionic imbalance caused by scaffold **7** induces autophagy that leads to onset of apoptosis in the parasite evident by the loss of mitochondrial membrane potential (ΔΨm) and DNA degradation. Our study provides a novel strategy for drug discovery and an insight into the molecular mechanism of ionic imbalance mediated death in malaria parasite.

## Introduction

The current anti-malarial chemotherapeutic regime against the deadly malaria parasite, *Plasmodium*, mainly comprises of artemisinin combinatorial therapy (ACT) that includes artemisinin and its derivatives combined with companion drugs such as amodiaquine, mefloquine, sulfadoxine-pyrimethamine (SP) and lumefantrine^1,2^. Most commonly used combinations are artemether-lumefantrine and artesunate-amodiaquine in African regions, dihydroartemisinin-piperaquine in South Asian countries and artesunate- sulfadoxine-pyrimethamine combination in India and some African countries^2^. However, malaria continues to pose a threat to human health given the increasing incidences of drug resistance against the current anti-malarial therapy^3,4^. Resistance against the currently available first-line of treatment for malaria calls for a more radical approach for the development of new anti-malarials.

Literature suggests that natural products have played an important role in the discovery of lead compounds for the development of drugs against human diseases including malaria^5^. Alkaloids are one of the major classes of nature derived compounds that exhibit antimalarial activity, Quinine being the best example of this class of compounds. Indole based natural alkaloids viz. Usambarine, 10′-hydroxyusambarensine, 18-hydroxyisosungusine, Sungusine, Naucleoffecines A, Naucleidinal, etc. are another examples of successful alkaloid based chemotherapeutics obtain from sub-tropical and tropical regions^6–8^.

In 2010, Rottman *et al*. reported the discovery of an indole based antimalarial agent KAE609 (cipargamin or NITD609), which is currently under phase II clinical trial (with sub-nanomolar potency against blood stage malaria parasite)^9^. KAE609 is more effective than the current gold standard treatment, Artemisinin against asexual blood-stage *P*. *falciparum* and is also capable of blocking transmission to mosquitoes^10^. In another example the Center for Chemical Methodology and Library Development at Boston University (CMLD-BU) discovered a scaffold from a collection of indole based natural products that proved to be an ideal motif for malaria-growth inhibition^11^. Several analogs of this scaffold exhibited low micro molar activity against five malaria strains.

Chiral bicyclic lactams popularized by Meyers *et al*. are a versatile class of molecules that essays the role of a chiral auxiliary as well as a building block^12–16^. Innumerable synthetic methodologies leading to several natural products have been documented based on these bicyclic lactams^17,18^. The key reactions involved either enantioselective base directed enolate chemistry on the methylene group alpha to the amide carbonyl that culminates into addition, arylation and cyclization or *via* thio-Claisen rearrangement of the corresponding thiolactams^19,20^.

Enantiomers with same chemical structure exhibit marked differences in their biological activities upon interactions with enzymes, proteins, receptors, etc. inside the body^21^. One isomer may be responsible for producing the desired therapeutic effect, while the other may cause toxicity or be inactive. Many drugs in market come in racemic mixture. Some of the examples of racemic drugs with one enantiomer as the major bioactive isomer are cardiovascular drug such as S(−)-propranolol which is 100 times more potent than its R(+)-isomer and calcium channel agonist, S(−)-verapamil which is 10–20 times pharmacologically more active than R(+)-verapamil^22–26^. Another important aspect of chirality is target specificity. One enantiomer may fit better into the catalytic/binding pocket than the other and may account for enhanced selectivity for biological targets, resulting in improved therapeutic indices and better pharmacokinetics than using a mixture of enantiomers.

Most of the current promising anti-malarials in pipeline belonging to the class of spiroindolones, aminopyrazole, etc. cause parasite death via disruption of ionic balance primarily by causing Na^+^ influx in the parasite^27,28^. This is achieved by disturbing the function of a P-type ATPase, PfATP4. PfATP4 contains the highly conserved acidic motif which is required for transport of Na^+^-ions in Na^+^-efflux ATPases (ENAs) present in lower eukaryotes including some protozoan which strongly supports the role of PfATP4 as ENA in the malaria parasite.

The mechanism of death stimulated by ionic imbalance is poorly understood in *P*. *falciparum*. Here we report a new and more effective drug designing strategy against the malaria parasite involving development of novel natural product inspired indole-based antimalarial scaffold, which induces cell death via ionic imbalance. Our potent scaffold disturbs Na^+^ homeostasis causing Na^+^ influx that induces the process of autophagy in the parasite. On further investigation we observed the classical features of apoptosis in the treated parasite, loss of mitochondrial potential and DNA fragmentation suggesting apoptotic type of cell death in the malaria parasite.

## Results

### Synthesis of potent antimalarial scaffolds using chiral bi-cyclic lactams as the building block

**Figure 1:**
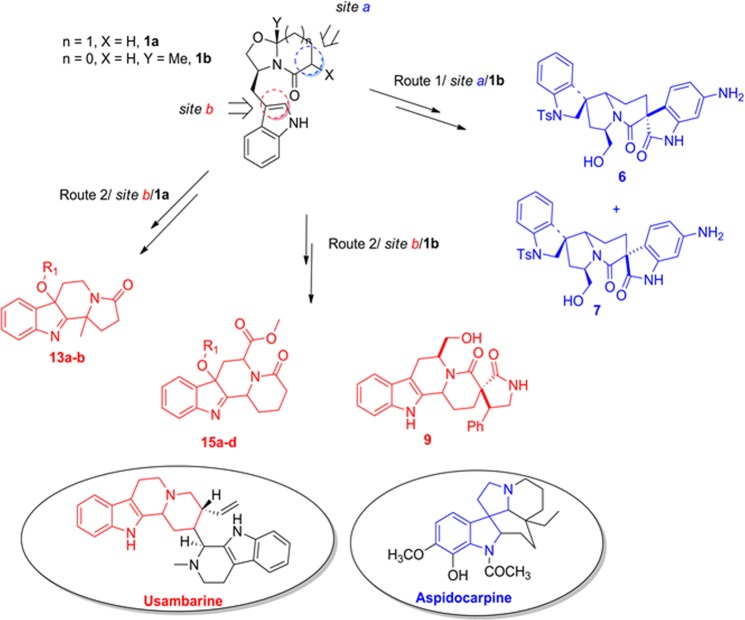
Overview of the synthesis strategy for natural compounds inspired scaffolds.

We envisioned a library inspired from antimalarial indole natural products Usambarine and Aspidocarpine (indolo [2, 3-*a*] quinolizidine and spiro [indole-3,3′-indolizoidine] class of alkaloids respectively) by using chiral bicyclic lactams **1**–**3** as building blocks (Scheme 1). We intended site-specific functionalization of **1**–**3** at sites a and b, which are the two most reactive sites that can be harnessed to generate the library (Fig. 1). Two routes were followed, wherein route 1 involved functionalization of **1b** and transformation of the bicyclic lactam thus obtained into Usambarine and Aspidocarpine scaffolds, and in route 2 it is conversion of the appropriate bicyclic lactams **1a**–**c** to the Usambarine scaffold and its five membered homologue followed by their functionalization (Fig. 1). The functionalities ranged from simple alkoxy and aryl moieties to privileged scaffolds like pyrrolidines and oxoindoles (Fig. 1).

**Figure 2:**
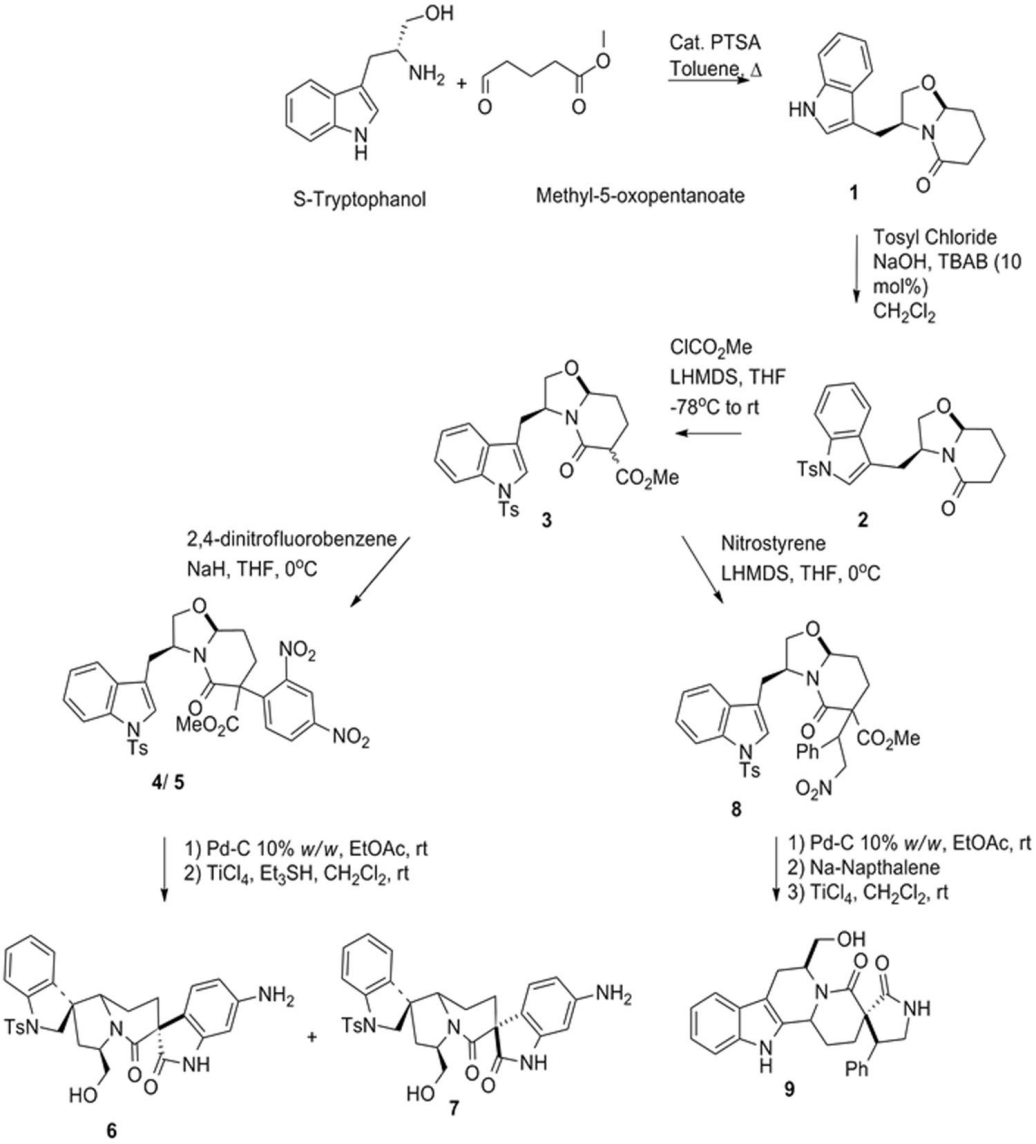
Synthesis of compounds: **6**, **7** and **9**.

To begin with, we targeted scaffolds **6**, **7**, and **9**, which we envisioned from **1** through site a/route 1. The synthesis of **1b** was achieved by following a literature procedure involving condensation of 5-methyloxopentanoate and S-tryptophanol in presence of catalytic *p*-toluenesulfonic acid with toluene as solvent^29^. In a bid to enrich **1b** with necessary functionalities N-tosylation of **1** led to the formation of **2**. Subsequent acetylation of **2** generated **3** as an equimolar mixture of its epimers. Aromatic nucleophilic substitution (S_N_Ar) with 2,4-dinitrofluorobenzene and michael addition with nitrostyrene diverged **3** into a mixture of **4/5** and **8** respectively. Hydrogenation of **4**/**5** with 10% *w/w* Pd-C and H_2_ at room temperature (RT) followed by TiCl_4_ and triethylsilyl hydride based spirocyclization of the subsequent intermediate generated **6** and **7**, which were purified by preparatory HPLC (Fig. 2). A similar hydrogenation of **8** followed by detosylation with sodium/naphthalene and TiCl_4_ based 6-endo-trig cyclization led to the formation of **9**. The relative stereochemistry of these compounds was confirmed by NOESY experiments. Hence this sequence afforded three scaffolds **6**, **7** and **9** from readily available starting materials with ample diversification and excellent steps per scaffold ratio of 2:3.

**Figure 3:**
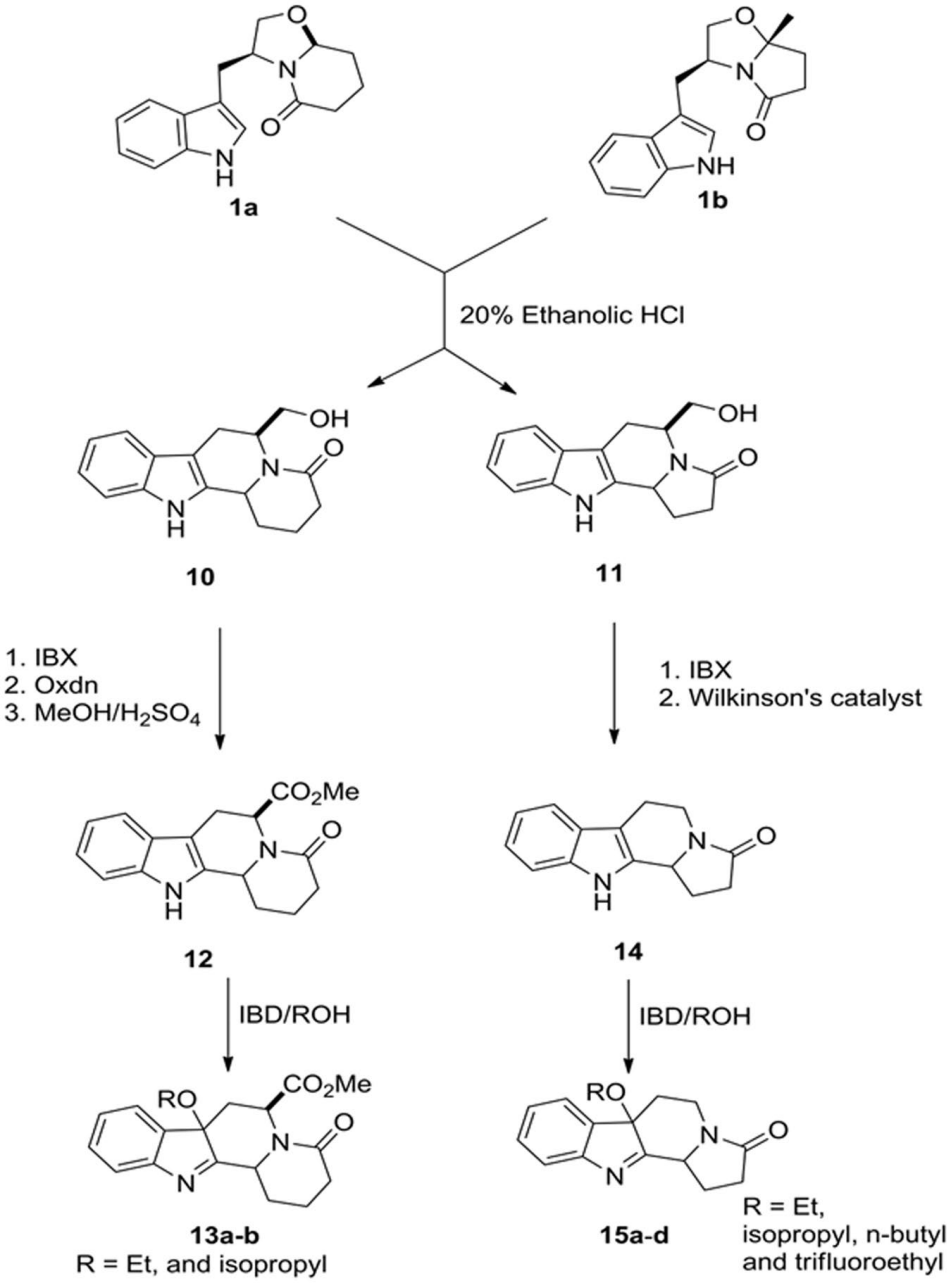
Synthesis of compounds **13a**,**b** and **15a**–**d**.

The next set of scaffolds was prepared via route 2/site b. Following a literature procedure chiral bicyclic lactams **1a** and **1b** were treated with ethanolic hydrochloric acid to get converted to fused scaffolds **10** and **11** in quantitative yield. Oxidation of **10** to carboxylic acid followed by decarboxylation afforded **12**. Parallel reaction of **12** in methanol, ethanol, n-butanol, isopropanol and trifluoroethanol in presence of DIB afforded compounds **13a–e**. In a similar fashion, **11** was oxidized to the corresponding carboxylic acid, which was esterified to afford **14**. A similar parallel reaction of **14**, with methanol, isopropanol, n-butanol and ethanol afforded **15a–d** (Fig. 3).

### Screening of indole based scaffolds library against ***in-vitro*** growth of *Plasmodium falciparum*

**Figure 4:**
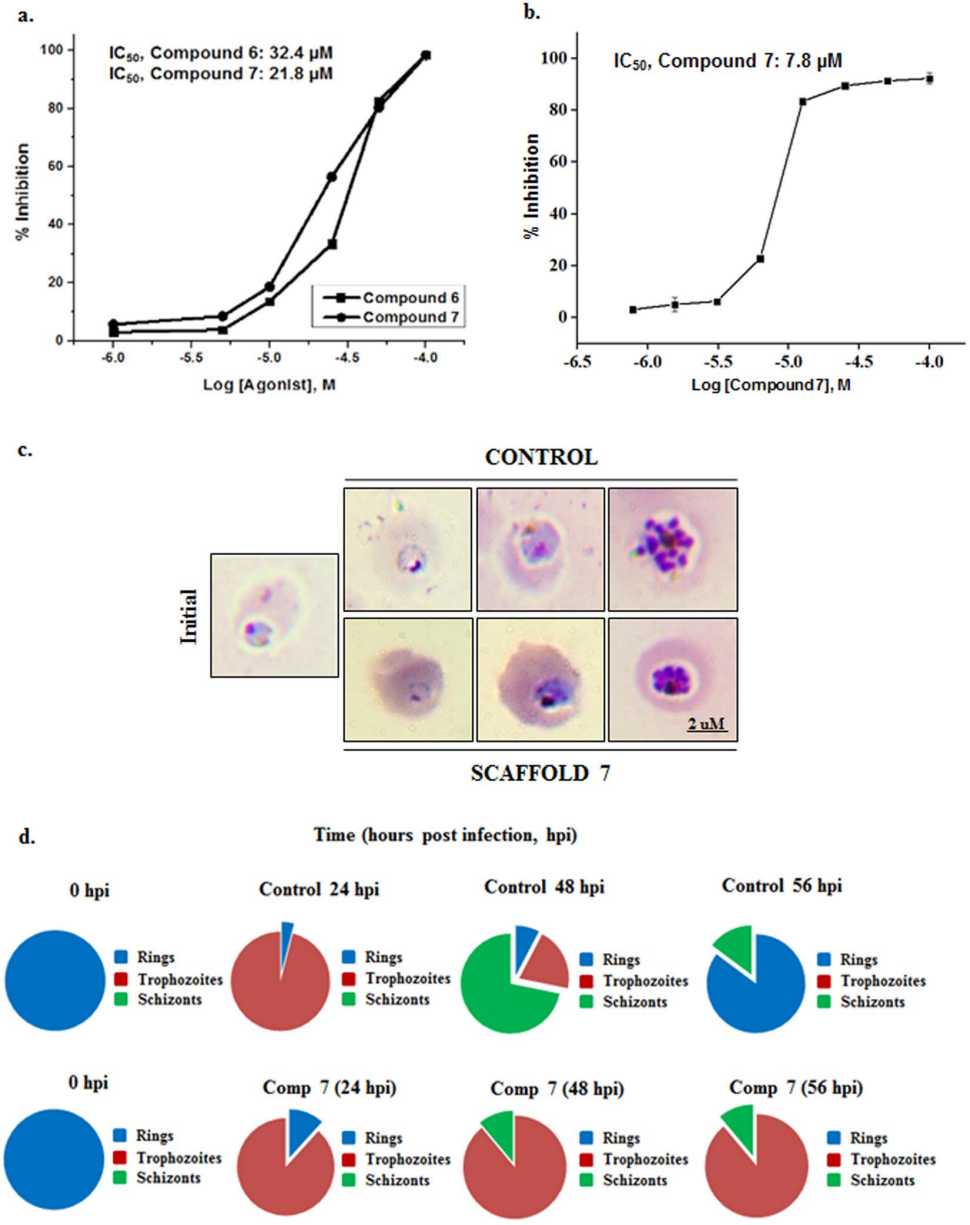
Inhibition of *Plasmodium falciparum* intra-erythrocytic growth. Ring stage parasites were treated with different concentrations (1–100 µM) of compounds 6 and 7. (**a**) Dose dependent effect of scaffolds **6** and **7** on parasite growth over one complete intra-erythrocytic life cycle of the parasite. (**b**) Dose dependent effect of scaffold **7** on the parasite growth over two consecutive intra-erythrocytic life cycles. (**c**) Microscopic images of *P*. *falciparum* culture incubated with IC_50_ concentration of scaffold **7**. In treated parasite, growth cycle got arrested at trophozoite stage. (**d**) Pie diagrams showing the distribution of parasite in different stages of the cycle at each time point. Spiro scaffold **7** halted parasite growth at the feeding stage, i.e. Trophozoites.

Scaffold screening is advantageous as it gives a good starting material to build a library of more potent chemotypes. Therefore representative members of the scaffold library such as 6, 7, 9, 13a, 14 and 15a were initially screened for their growth inhibitory effect, if any, at standard concentration of 50 μM over one complete intra-erythrocyte life cycle of the parasite (Table 1). Scaffold **7** and its diastereoisomer **6**, showed more than 80% growth inhibition of the parasite. Two hits obtained after initial phenotypic screening were further subjected to dose response over one complete intra-erythrocyte parasite growth cycle with six concentrations: 1, 5, 10, 25, 50 and 100 μM (Fig. 4a). Half maximal inhibitory concentration i.e. IC_50_ was determined for scaffold **6** and **7** and was found to be 32.4 μM and 21.8 μM respectively. Scaffold 7, with lower IC_50,_ was further accessed for parasite growth inhibition activity over two consecutive rounds of egress and invasion, thereby lowering down the IC_50_ to 7.8 μM (Fig. 4b). In both of the cases, untreated parasites were taken as control and percent growth inhibition was evaluated by flow cytometry, as described in methods. Scaffold **7** was tested for its cytotoxicity on mammalian cell line, HepG2 at concentration double the IC_50_ of scaffold **7** i.e. at 50 μM. Treated HepG2 cells were as healthy as control cells (Supplementary Fig. 1). In addition, scaffold **7** was found to be non-toxic even at milli-molar level in mammalian cell lines, HepG2 and COS7.

**Table 1:** Intra-erythrocytic growth inhibition of *P*. *falciparum* by indole based scaffold library.

S. No.	Manuscript ID	Structure	Mol. Wt.	% inhibiton at 50 µM (3D7)
1	6		558.65	83 +/− 4
2	7		558.65	80 +/− 4
3	9		415.48	3 +/− 2
4	13a		847.01	13 +/− 1
5	14		460.56	21 +/− 2
6	15a		851.04	10 +/− 2

Unless indicated the differences were considered to be statistically significant at P < 0.05.

### Effect of potent chiral scaffold 7 on progression of blood stage growth cycle of *Plasmodium falciparum*

Biological systems recognize two enantiomers or diastereoisomers as two distict molecules, and their interaction therefore elicits different responses. Scaffold **7** was found to be more potent with a lower IC_50_ than scaffold **6**. Stage specific effect of **7** at its IC_50_ concentration was examined by analyzing the morphology of treated parasites at 12, 32 and 42 hours post infection (hpi). Nearly 5,000 cells/slide at each time point were counted in duplicates. In control, the parasites were healthy and completed its life cycle producing rings at 48 hpi. But in treated culture, ring formation was not observed. More than 90% of the parasites were stalled at early/mid trophozoites stage (Fig. 4c). This clearly indicated that the parasite could not complete its growth cycle in the presence of diastereoisomer **7** and the growth was halted at trophozoite stage, as represented in the pie diagram (Fig. 4d).

### Scaffold 7 disturbs Na^+^ homeostasis in *Plasmodium falciparum*

**Figure 5:**
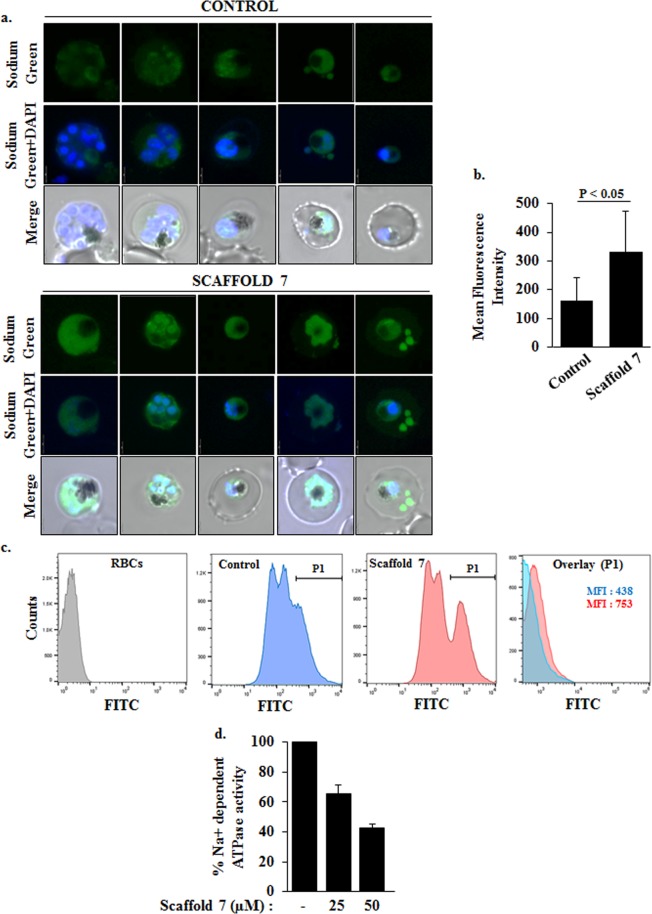
Scaffold **7** causes Na^+^ influx in *P*. *falciparum* by targeting a Na^+^ dependent ATPase. (**a**) Representative confocal microscopic images of *P*. *falciparum* stained with Sodium Green showing rise in Na^+^ levels in the treated parasites. Scaffold **7** increases the cytosolic concentration of Na^+^. (**b**) Bar graph depicts the mean fluorescence intensity of sodium green in control and treated parasites. (**c**) Flow cytometry data to support elevation of intracellular Na^+^ in Scaffold 7 treated parasites. (**d**) Inhibition of Na^+^ dependent ATPase activity in membrane fraction of parasite. We observed around 40% to 60% inhibition in Na^+^ dependent ATPase activity in scaffold **7** treated parasite.

In other eukaryotes it is known that ionic imbalance mediated by various cation exchangers leads to mitochondrial dysfunction eventually culminating in apoptotic cell death^30,31^. Role of cations such Na^+^ individually or in coherence with other ions is also implicated in stimulation of cell death^32–36^. Interestingly, the upcoming anti-malarials in pipeline have been reported to induce parasite death by causing a rise in cytosolic Na^+^ concentration, [Na^+^], creating Na^+^ imbalance^10,37–39^. However in *P*. *falciparum*, the understanding of ionic imbalance mediated parasite death is very rudimentary. So we looked for changes in intracellular [Na^+^] in the parasite post treatment with the potent scaffold. To our interest, based on confocal microscopic imaging of *P*. *falciparum* stained with Sodium Green, scaffold **7** treated parasites showed an increase in cytosolic [Na^+^] as compared to control parasites (Fig. 5a). The mean fluorescence intensity of sodium green in control and treated parasites was calculated to quantify the elevation in [Na^+^] in treated parasite (Fig. 5b). Confocal imaging data was further confirmed by analyzing flow cytometry data (Fig. 5c).

### Scaffold 7 targets membrane ATPases of *Plasmodium falciparum*

To test directly whether the potent indole-based scaffold **7**, which is a spiroindolone, inhibit Na^+^ dependent membrane ATPases in the parasite, we investigated ATPase activity in membrane preparations of the purified trophozoites. Firstly, we isolated the membrane fraction from un-treated and scaffold **7** treated parasites and then evaluated the ATPase activity using Malachite Green based assay, as described in materials and methods section. In the whole membrane ATPase pool of *Plasmodium falciparum*, we found that Na^+^ dependent ATPases contribute 33.4% +/− 4.34% activity. Therefore, Na^+^ dependent ATPase activity contributes a significant fraction of the total membrane-associated ATPase activity in parasitized RBCs. Upon treatment of isolated membrane fraction of the parasite with scaffold **7** in the presence of 100 mM Na^+^, the Na^+^ dependent ATPase activity declined to ~65.6% ± 5.6% (25 µM) and ~42.4% ± 2.8% (50 µM) with respect to control (Fig. 5d). However, treatment of membrane fraction with scaffold **7** in the low (0.5 mM) [Na^+^] solution did not alter the ATPase activity significantly (Supplementary Fig. 2), thereby implying that the spiroindolone (scaffold **7**)-sensitive ATPase activity was present under high- [Na^+^] conditions.

### In silico interaction of scaffold 7 and its diastereoisomer, scaffold 6 with PfATP4

**Figure 6:**
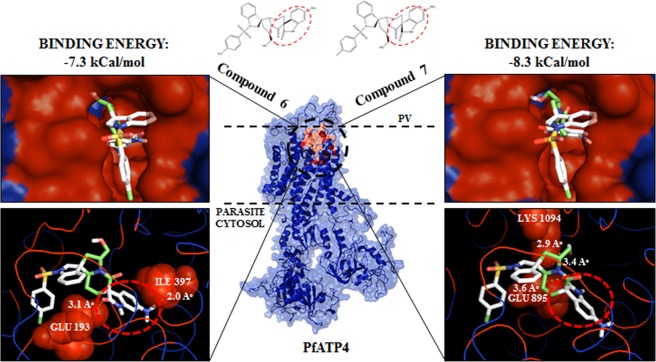
Homology model of PfATP4 three-dimensional structure and insights into the molecular interactions with scaffolds **6** & **7**. Homology model of ATP4 was generated using MODELLER taking rabbit Ca^2+^ ATPase (PDB ID: 2DQS) as template (middle). Chemical structures of compounds 6 and 7 and their energy minimized models were generated by using ChemDraw Ultra 12.0.2 and Chem3D Pro 12.0 respectively. Molecular docking studies with PfATP4 and scaffolds **6** & **7** performed using Autodock Vina Tools 1.5.6. (**a**,**b**) Both scaffolds fit into the predicted PfATP4 active binding pocket (red). (**c**) Binding modes of scaffold **6** in the binding pocket. GLU-193 and ILE-397 of ATP4 were found to interact with scaffold **6** via polar interactions (H-bonds, indicated by dashed lines) with bond lengths, 3.1 Å and 2.0 Å respectively (left). (**d**) Binding modes of scaffold **7** in the binding pocket. GLU-895 and LYS-1094 of ATP4 were found to interact with scaffold **7** via H-bonds with bond lengths 3.6 Å and 3.4 Å, 2.9 Å respectively (right). Change in chirality allows for scaffold **7** to reasonably fit into the binding pocket with more negative free binding energy (−8.3 kCal/mol) as compared to scaffold **6** (−7.3 kCal/mol). Three-dimensional visualization of protein structure and interactions analysis was rendered with PyMOL Molecular Graphics System, Version 1.7.4.5 Schrödinger, LLC.

*P*. *falciparum* maintains a low cytosolic [Na^+^] environment which is mediated by a P-type Na^+^-ATPase ‘PfATP4’ localized on surface of the parasite^40^. In recent years, PfATP4 has gained attention as mutations in this protein have been reported to confer resistance to the new classes of antimalarial compounds including spiroindolones, pyrazoles, dihydroisoquinolones and many more, suggesting PfATP4 as a critical target to develop new antimalarials^27,28^. Diastereoisomers **6** and **7** were found to fit into overlapping binding pockets of PfATP4 (Fig. 6) and interact through polar contacts (H-bonds) with amino acid residues constituting active site of the protein. But at the same time, change in chirality allowed for scaffold **7** to reasonably fit better into the PfATP4 binding pocket, with more negative free binding energy (−8.3 kCal/mol) as compared to scaffold **6** (−7.3 kCal/mol), which was reflected in the difference in IC_50_ values of the two scaffolds. Scaffold **7** was found to form three H-bonds with GLU-895 (3.6 Å) and LYS-1094 (3.4 Å), and LYS-1094 (2.9 Å).

### Ionic imbalance caused by scaffold 7 induces autophagy in *Plasmodium falciparum*

**Figure 7:**
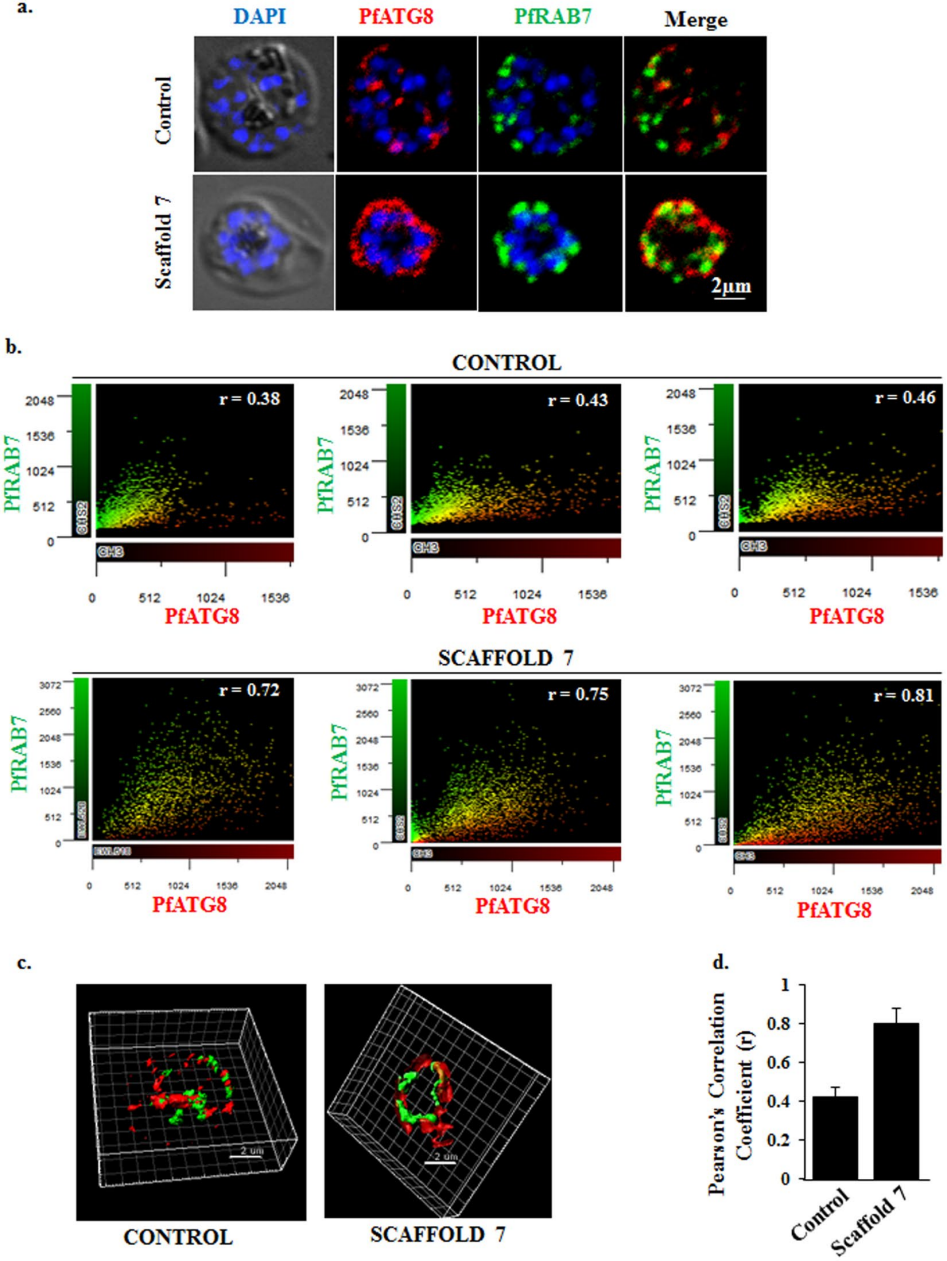
Ionic imbalance initiated autophagy leads to apoptosis in *Plasmodium falciparum*, thereby inducing co localization of PfATG8 and PfRAB7. (**a**) Fluorescent microscopy images of sub-cellular distribution of PfATG8 and PfRAB7 in schizonts stained with anti-PfATG8 rabbit sera and anti-PfRAB7 rat sera. Nuclear DNA was counterstained with DAPI. In scaffold 7 treated parasite, PfATG8 co-localizes with PfRAB7 indicating parasite is undergoing autophagy. (**b**) Co-localization analysis from scatter plots in support of the confocal images. Representative scatter plots and mean Pearson’s correlation coefficients (r) from both control and treated group of parasites were analyzed by using Olympus cellSens dimensions imaging software. Red channel (PfATG8) is shown on the x-axis and green channel (PfRAB7) is shown on the y-axis. The yellow dots on scatter plots show the co-localization. (**c**) Three-dimensional reconstruction of confocal z-stack images of both the control and treated parasites, as acquired by Imaris - microscopy image analysis software. PfATG8 is represented as red and PfRab7 as green. Treatment of the parasites with scaffold 7 results in colocalization of ATG8 and RAB7. (**d**) Colocalization of PfATG8 and PfRAB7 in treated parasites was quantified by calculating Pearson’s correlation coefficients (R), represented in the form of a bar graph.

ATG8 is considered as the classical marker of autophagy because of its association with pre-auto phagosomal membrane as well as during fusion of auto-phagosome with lysosome. This process is mediated through RAB7 and various SNAREs^41,42^. Recently, Tomlins *et al*. reported that under stress or starvation condition in blood stage parasite, PfATG8 co-localizes to double-membrane structures/vesicles with PfRAB7 located near or inside the food vacuole indicating autophagy^43^. To determine if scaffold **7** induced autophagy, parasites were treated with scaffold **7** and the sub-cellular distribution of PfATG8 and PfRAB7 was observed using anti-sera raised against recombinant ATG8 and RAB7 protein. In control, distribution of the two proteins overlapped partially, while in treatment co-localization was observed (Fig. 7a). Representative scatter plots and mean Pearson’s correlation coefficients from both control and treated group of parasites: *r*_*control*_ = 0.42 ± 0.05, n = 25; *r*_*treated*_ = 0.80 ± 0.08, n = 25 are shown; red intensity (PfATG8) is shown on the x-axis and green intensity (PfRAB7) is shown on the y-axis. The yellow dots on scatter plots show the co-localization (Fig. 7b,d). 3D images from both the control and treated parasites as acquired by Imaris - microscopy image analysis software are shown which clearly indicates that treatment of the parasites with scaffold **7** results in colocalization of ATG8 and RAB7 (Fig. 7c). From this experiment it was speculated that scaffold **7** via disturbing the ionic balance in the parasite’s environment induces autophagy.

### Scaffold 7 induced autophagy triggers apoptosis via loss of mitochondrial membrane potential

**Figure 8:**
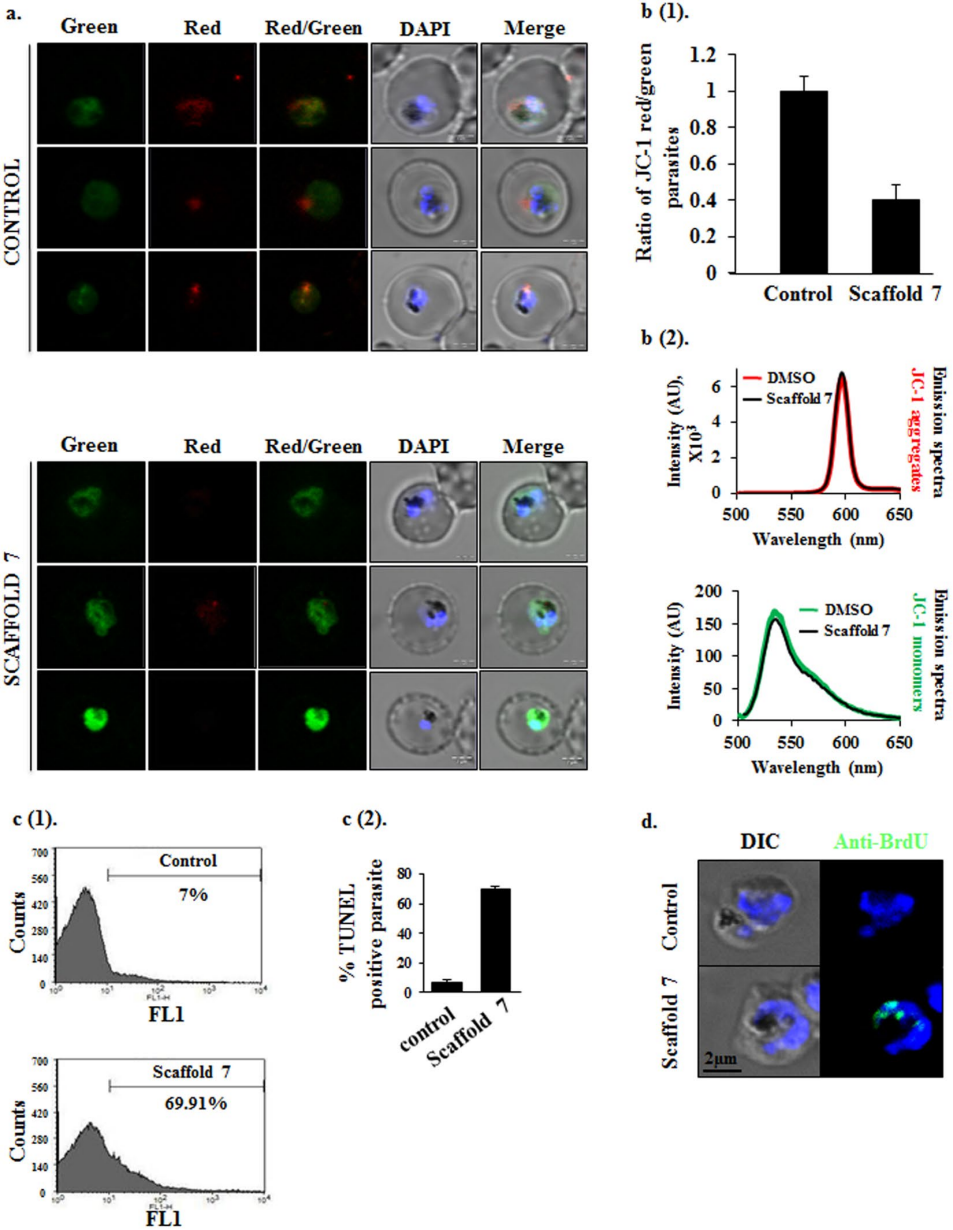
Loss of mitochondrial membrane potential and apoptosis like cell-death in scaffold-7 treated *Plasmodium falciparum* parasites. (**a**) Representative confocal microscopic images of *P*. *falciparum* infected RBCs stained with JC-1. Loss of mitochondrial membrane potential indicated by more green fluorescence of JC-1 monomers in parasite treated with scaffold **7** while in control JC-1 aggregates gives an intense red fluorescence. (**b**) (1) Bar graph depicts the ratio of JC-1 red/JC-1 green parasites in control and treatment. Treated parasite population exhibits low ratio of JC-1 red/JC-1 green indicating loss of mitochondrial membrane potential caused by scaffold **7**. (**b**) (2) Fluorescence emission spectra at 488 nm indicates that scaffold **7** does not interfere with JC-1 aggregation in a cell free system. (**c**) (1) Flow cytometry histograms showing increase in proportion of TUNEL positive parasite (FL1) after scaffold **7** treatment, signifying DNA fragmentation. (**c**) (2) Representative bar graph demonstrating percentage population, which is TUNEL positive. (**d**) TUNEL staining of parasites shows *in-situ* DNA fragmentation after incubation with scaffold **7**. AU: Arbitrary Unit.

When damage to a cell is beyond repair, autophagy often leads to apoptosis. To conclude the type of death induced by scaffold **7** we studied the classical events of apoptosis in treated parasite. Loss in mitochondrial membrane potential (Ψ_m_) is one of the key phenomenon for parasite undergoing apoptosis^44^. Accordingly, any change in Ψ_m_ of parasites treated with scaffold **7** was investigated via JC-1 staining. JC-1 is a cell-permeable lipophilic dye that exhibits Ψm-dependent aggregation in mitochondria as indicated by its fluorescence emission shift from green (~529 nm) to red (~590 nm)^45^. High Ψm of a healthy mitochondria results in the formation of JC-1 aggregates which give an intense red fluorescence while loss in Ψm results in a weak red fluorescence. Hence to assess the loss of Ψ_m_, the ratio of JC-1 (red)/JC-1 (green) parasites was calculated. In control parasites, healthy mitochondria showed strong red fluorescence of JC-1 aggregates with diffused green fluorescence (JC-1 monomers) in the parasite’s cytoplasm. On the contrary, only green fluorescence was observed in parasites treated with **7** relative to control, suggesting loss of Ψ_m_ (Fig. 8a). Overall, following treatment, low ratio of JC-1 (red)/JC-1 (green) parasites, i.e., 0.4 for scaffold **7**, indicated loss in mitochondrial membrane potential (Fig. 8b(1)). This conclusion was further strengthened by observing that scaffold **7** does not interfere with JC-1 aggregation by measuring fluorescence emission spectra of JC-1 (2 μM) at excitation wavelength of 488 nm in a cell-free system, in the absence and presence of scaffold **7**. We noticed that in aqueous media containing <1% DMSO (*in vitro* condition in which almost all JC-1 is present as aggregates), 488 nm-excited JC-1 emits at 595 nm with similar emission intensity in the absence and presence of scaffold **7**, indicating that scaffold **7** does not interfere with JC-1 aggregation. Simultaneously, in the presence of 35% DMSO (condition in which all JC-1 is present as monomers), JC-1 emission peaks at 530 nm equally in the absence and presence of scaffold **7** (Fig. 8b(2)).

### DNA damage triggered apoptosis like cell death in *P*. *falciparum*

DNA degradation is a classical marker of apoptotic death in a cell. TUNEL assay is the gold standard method for detection of the fragmented DNA^44^. This assay is based on labeling of 3′-OH termini of DNA strand breaks by incorporation of BrdU using terminal deoxynucleotidyl transferase (TdT). Similarly, in *P*. *falciparum*, DNA fragmentation is an indicator of apoptosis, which has been demonstrated via TUNEL assay using flow cytometry method^46,47^. An alternate method, DNA ladder assay is also often used to exhibit DNA degradation during apoptosis. However, it has been reported in earlier studies that the malaria parasites are devoid of histone variant H2AX homologue required for characteristic formation of DNA ladder^48^. Moreover, no true caspases have been reported in the parasite. Instead, caspase-like activity (metacaspases) has been described during apoptosis^49^. Therefore, genomic DNA degradation is observed as a smear instead of typical laddering pattern. Thus, TUNEL assay is the most reliable assay to show apoptosis in *P*. *falciparum*.

We performed DNA fragmentation studies in treated and untreated parasites by TUNEL assay using APO-BrdU™ TUNEL Assay Kit (Molecular Probes, Invitrogen). This assay allows *in-situ* labeling of fragmented DNA. The labelled cells represent parasites undergoing apoptotic death. The population of labeled parasites was then analyzed through flow cytometry that allows analysis of large population of cells on a single cell basis to give more confirmatory results. We found that in treated parasites more than 60% of total parasite population was TUNEL positive indicating that treatment with scaffold **7** causes apoptotic death in *P*. *falciparum*. The flow cytometry data was plotted as histograms (Fig. 8c1). The results of three independent experiments (n = 3) was plotted in a bar graph that showed the percentage of distinct TUNEL positive population of parasites in scaffold **7** treated parasites (69.91%) and untreated parasites (7%), (Fig. 8c2).

To further strengthen our results, we analyzed the apoptotic population in treated and untreated parasites using confocal microscopy (Fig. 8d). In treatment, fragmented parasite DNA was clearly represented by the green fluorescence from Fluor^R^488 dye–labelled anti-BrdU antibody highlighting the process of apoptosis in parasites. In control, healthy parasites showed negligible green fluorescence.

## Conclusion

Indole-based anti-malarials have been successful irrespective of their source i.e. natural or synthetic^6–9^. Hence, the preponderance of indole-based antimalarial compounds prompted us to design a library of skeletally diverse indole-based compounds with high sp^3^ content, diverse shape and physical properties that could be potentially anti-malarial. Since a good starting scaffold is amenable to various modifications to generate more potent chemotypes, scaffolds screening offers an attractive alternative to the traditional high throughput screening of final library of compounds. Six structurally and stereo-chemically different natural alkaloids inspired scaffolds were evaluated for their efficacy against blood stage malaria parasite, *P*. *falciparum*.

In a chiral environment constituted by building blocks of life such as amino acids, sugars and lipids, one isomer may exhibit different biological and pharmacologic behavior than its enantiomer. Even the drug targets are often enantioselective towards their binding partner^21^. Therefore it is possible that one of the two enantiomers have a better binding into the active site pocket of the target resulting in better biological response. Two of the representatives of scaffold library, scaffold **7** and its diastereoisomer **6** with spiro [indole-3,3′-indolizoidine] motif exhibited efficacy against *3D7* strain of malaria parasite. Scaffold **7** with IC_50_ in low micro-molar range (21.8 µM) and no cytotoxic effect on mammalian HepG2 was investigated further for its mechanism of action.

In the conventional drug discovery approach, cellular IC_50_ of hit compounds in nano-molar range have been considered as clinically useful concentrations. However, literature mining and overview of marketed FDA approved drugs indicates the existence of widely used compounds which are effective at low micro-molar range, with no cytotoxic effects on human cell lines at even high concentrations. These pharmacological agents are either administered individually or as a part of combinatorial drug therapy in a combination of two or more active ingredients in a single-dosage formulation. One of the early evidences comes from a study in which IC_50_ for pyrimethamine, a widely accepted anti-parasitic compound used in the treatment of chloroquine resistant *P*. *falciparum* malaria, was reported to be greater than 10 µM^50^. Another evidence comes from human adrenal steroid hormone Dehydroepiandrosterone (DHEA) analogue, DHEA-Sulfate (DHEA-S), which exhibits anti-malarial activity against chloroquine-sensitive strains of *P*. *falciparum* at IC_50_ of 19 µM, by enhancing phagocytosis of ring stage parasitized RBCs^51^. Temporal monitoring of *in-vitro* susceptibility of *Plasmodium falciparum* to doxycycline, another widely used antimalarial compound, demonstrated it’s mean IC_50_ to be 9.6 µM in 1996 to 1999, and 13.1 µM in 2005^52^. Similarly, azithromycin, an azalide analog of erythromycin, which is efficacious against *Plasmodium vivax* malaria, shows IC_50_ value of 8.4 μM against multidrug-resistant *P*. *falciparum K1* strain^53^.

On similar lines, although scaffold **7** has IC_50_ in low micro-molar range yet it holds a great promise as a potential anti-malarial chemotherapeutic both as an independent drug and in partnership with other drugs. Moreover it comes with an enormous scope for deriving better potent lead compounds by substitution of groups around scaffold **7**.

Scaffold 7 targets Na^+^ balance in the parasite in a similar fashion as do the next line of anti-malarials in pipeline. Post invasion *P*. *falciparum* establishes new permeability pathways in the host RBC membrane for nutrient uptake into the infected cell^54–56^. Along with nutrients there is Na^+^ influx, resulting in increasing [Na^+^] in the infected erythrocyte cytosol to high levels. The intra-erythrocytic parasite however maintains a low cytosolic [Na^+^]^40^. The plasma membrane P-type cation translocating ATPase ‘PfATP4’ plays a crucial role in this process. Thus, PfATP4 has emerged as the most promising target for current classes of anti-malarial compounds such as spiroindolones, pyrazoles, dihydroisoquinolones, etc^27,28^. It seems to be the possible target for our potent scaffold 7 evident by the molecular docking of scaffold **7** into the PfATP4 binding pocket. Furthermore, inhibition of Na^+^-dependent ATPase activity in parasite membrane fraction treated with scaffold 7 shows that it targets PfATP4 located in the parasite plasma membrane, which causes Na^+^ influx in the parasite which otherwise maintains a low [Na^+^] in its cytosol. Despite being the most potential and favorable target for the next generation anti-malarials including our scaffold **7**, the type of death induced by the ionic imbalance created by disruption of PfATP4 is however not known until now.

The two processes known to maintain overall cellular homeostasis i.e. apoptosis (self-killing) and autophagy (self-eating) have a complex functional inter-relationship. They may be activated by common signals, which may result in combined autophagic and apoptotic processes. Ionic imbalance often initiates autophagy in a cell. In autophagy, auto-phagosome formation is the central event and is mediated by autophagy related proteins (Atg). One of the Atg proteins, Atg8 is considered as the classical marker of autophagy because of its association with pre-auto-phagosome membrane as well as during fusion of auto-phagosome with lysosome. Fusion of both auto-phagosomes and endosomes with the lysosome is mediated through RAB7 and SNAREs (N-ethylmaleimide-sensitive factor attachment protein (SNAP) receptors)^41,42^. In 2013, Tomlins *et al*. reported that under starved conditions in blood stage parasite PfATG8 co-localizes to double-membrane structures/vesicles with PfRAB7 located near or inside the food vacuole indicating onset of autophagy. We found a similar staining pattern in our scaffold **7** treated parasites^43^. The most common cause of death reported in *Plasmodium* is apoptosis. Loss of Ψm and DNA degradation are two most common features of apoptotic death. We studied these two classical features of apoptosis in scaffold **7** treated parasites. JC-1 dye that exists in monomeric form or as aggregate depending on membrane potential of mitochondria gave a dispersed intense green fluorescence in the treated parasite indicating loss of Ψ_m_. To study DNA fragmentation we performed TUNEL assay, the most preferred method to demonstrate DNA damage. In *P*. *falciparum*, DNA fragmentation is considered as a hallmark of apoptosis and has been studied by using TUNEL assay^46,47^. Given the absence of histone variant H2AX homologue and true caspases in *P*. *falciparum*, which are needed for the formation of characteristic DNA ladder, degraded genomic DNA is observed as a smear than the classical ladder pattern^48,49^. Thus, TUNEL assay stands out as the best method to demonstrate apoptosis in *P*. *falciparum*. We, therefore, performed the gold standard assay for DNA fragmentation, TUNEL, with scaffold **7** treated parasites and observed that more than 60% of total treated parasite population to have undergone DNA degradation suggesting that scaffold **7** induces apoptosis like cell death in *P*. *falciparum*.

**Figure 9:**
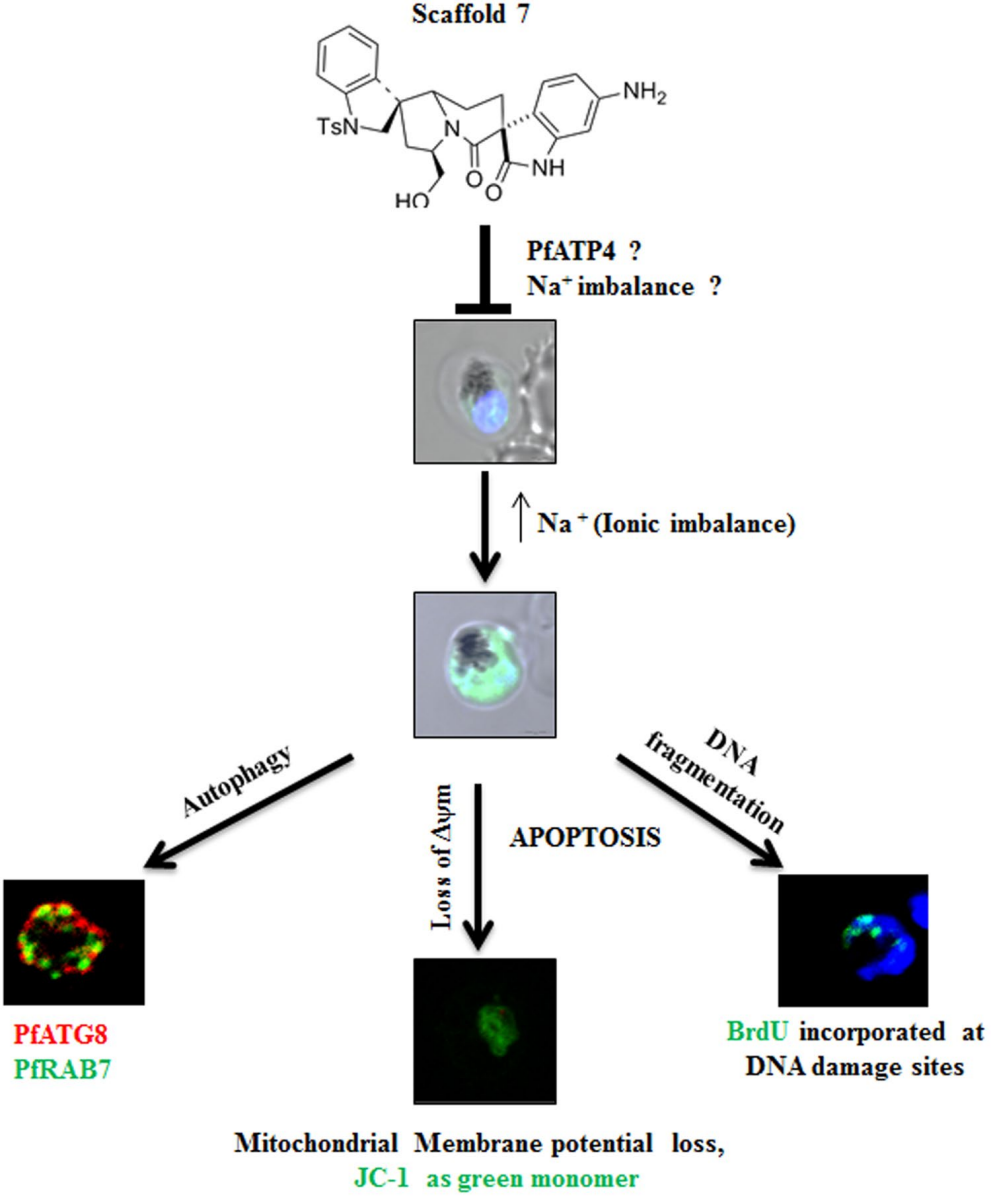
Ionic imbalance mediated apoptotic death in malaria parasite. Scaffold **7** causes Na^+^ influx that induces autophagy, which stimulates apoptotic death in parasite. PfATP4, a Na^+^ dependent ATPase could be a potential target of scaffold **7**.

To conclude, we present a novel natural product inspired drug-designing approach that yielded us an indole based potent scaffold capable of dysregulating Na^+^ homeostasis in parasite. This study provides a significant contribution in understanding the ionic imbalance mediated cell death in parasite, which occurs via induction of autophagy and finally culminating in apoptosis (Fig. 9).

## Materials and Methods

### Biological assays

#### *In vitro* culturing of *P*. *falciparum* and mammalian cell line, HepG2

*Plasmodium falciparum* laboratory strain 3D7 used in this study was cultured in O^+ve^ erythrocytes at 2% haematocrit in RPMI 1640 medium (Invitrogen) supplemented with 0.5% Albumax I (Invitrogen), 50 mg/l hypoxanthine (Sigma), 10 mg/l gentamicin (Invitrogen) and 25 mM sodium bicarbonate (Sigma), as described by Trager and Jensen, and Singh, S. *et al*.^57,58^. Parasite culture was maintained in a mixed gas environment (5% O_2_, 5% CO_2_ and 90% N_2_). Cultures were tightly synchronized at ring stage by two successive rounds of sorbitol treatment, as previously described^58,59^.

The human liver carcinoma cell line, HepG2 was cultured in Dulbecco’s Modified Eagle’s Medium (Invitrogen, USA) supplemented with 10% FBS (Gibco, USA) and penicillin (100 units/ml) and streptomycin (100 mg/ml). All cultures were grown in culture flasks at 37 °C in a humidified atmosphere of 5% CO_2_.

#### Evaluation of growth inhibitory effect of novel indole based scaffolds

Six chemical scaffolds obtained were subjected to initial screening at concentration of 50 μM for their inhibitory effect on parasite growth. The two potent scaffolds were further subjected to dose dependent evaluation at concentrations ranging from 1–100 μM, untreated as control. Briefly, late-stage parasites at an initial parasitemia of 1% and 2% hematocrit were treated with compounds, as described by Kumar, N. *et al*.^60^. Post incubation, parasites were washed with PBS and stained with ethidium bromide (10 μM) for 30 minutes at RT in dark, as described by Sharma, V. *et al*.^61^. Following two washes with PBS, cells were analyzed by flow cytometry on BD LSRFortessa™ cell analyzer by using FlowJo v10 software. Fluorescence signal (FL-2) was detected with the 590 nm band pass filter using an excitation laser of 488 nm collecting 100,000 cells per sample. Following acquisition, parasitemia was estimated by determining the proportion of FL-2-positive cells using Cell Quest.

Growth inhibition (% Inhibition) was calculated as follows:

$$ \% \,{\rm{Inhibition}}=[1- \% \,{\rm{Parasitemia}}\,({\rm{Treatment}})/ \% \,{\rm{Parasitemia}}\,({\rm{Control}})]\,\ast \,100.$$

#### Statistical analysis

In the bar graphs, the data for the IC_50_ values and % parasite growth are expressed as the mean ± standard deviation (SD) of three independent experiments done in duplicates^61^. IC_50_ values were calculated using OriginPro Evaluation 2018b Graphing and Analysis software. Unless indicated, differences between the mean values were considered to be statistically significant at P < 0.05.

#### Growth progression assays

Effect of scaffold **7** on growth progression of parasite was assessed by treating parasite diluted to 0.5% parasitemia and 2% haematocrit at concentration equivalent to IC_50_ of the compound. Culture at ring stage (12–14 hpi) was treated in duplicate in a 96 welled plate with solvent as control. Monitoring of the parasite progression through its asexual blood stage was done at 24, 48 and 56 hpi. At every time point, Giemsa-stained thin blood smears of *P*. *falciparum* were made and around 5,000 red blood cells (RBCs) were counted by light microscopy^61^.

#### Cytotoxicity assay with HepG2 cell line

Cytotoxicity assay was performed in 96-welled micro-titer plates with HepG2 cells, seeded at a seeding density of 30,000 cells per well and were allowed to adhere overnight at 37 °C, as described previously^61^. HepG2 cells were treated with scaffold **7** at 50 μM concentrations for 24 hours. Cytotoxic effect was assessed using ability of live cells to cleave MTT ((3-[4,5-dimethylthiazol-2-yl]-2,5-diphenyl tetrazolium bromide)) (Sigma-Aldrich, St Louis, MO, USA), into formazan crystals, as described by Bathula, C. *et al*. and Hati, S. *et al*.^62,63^. The crystals were dissolved in DMSO (Dimethyl Sulfoxide, Sigma-Aldrich, St Louis, MO, USA). The colorimetric assay was read at 590 nm wavelength using spectrophotometer.

#### Measurement of intracellular Na^+^ level in *P*. *falciparum*

The intracellular sodium ion [Na^+^]_i_ concentration was measured at 37 °C by using the cell-permeant Na^+^ sensitive dye, Sodium Green (Molecular Probes, Invitrogen). Percoll purified parasites were resuspended in incomplete RPMI and loaded with 10 μM Sodium Green for 30 min at 37 °C. The dye-loaded parasites were washed twice with incomplete RPMI and treated with scaffold **7** for 4 hours. Post treatment, parasites were washed with 1XPBS. The excitation and emission wavelength for Sodium Green is 507 nm and 532 nm respectively. The labeled parasites were analyzed by confocal microscopy and flow cytometry.

#### ATPase activity in membrane fraction of *Plasmodium falciparum* in the presence of scaffold 7

ATPase activity contributed by membrane fraction of the parasite was evaluated by measuring the rate of production of Pi using Malachite Green Phosphate Assay Kit (BioAssay Systems, POMG-25H) that relies on estimation of green colored complex formed between Malachite Green, molybdate and free orthophosphate (PO_4_^3−^) released during the ATPase reaction(s). Protocol was followed as described previously^64^. Briefly, purified trophozoites were lysed by freeze-thaw in 0.1X PBS supplemented with protease inhibitor cocktail, followed by centrifugation at 13,000 rpm at 4 °C for 30 minutes. Pellet (membrane fraction), thus obtained, was then washed three times in Na^+^-depleted assay buffer (50 mM Tris-HCl, 20 mM K^+^, 2 mM Mg^2+^, 100 mM Na^+^) before its immediate use in the assay. Membrane fractions were resuspended in either high [Na^+^] solution (100 mM) or low [Na^+^] solution (0.5 mM), in the absence or presence of different concentrations (25 µM and 50 µM) of spiro scaffold **7**. Reactions were initiated by adding 0.25 mM ATP and incubated at 37 °C. Color formation in the reaction mixtures was measured after 20 minutes on a plate reader (655 nm).

#### Homology modeling of PfATP4

The sequence of *Plasmodium falciparum* cation ATPase, PfATP4 was obtained from PlasmoDB database (PF3D7_1211900). Homology modelling of PfATP4 three-dimensional structure was done by using MODELLER which is a program used for comparative modeling of protein structure(s) by satisfaction of spatial constraints^65–67^. Rabbit Ca^2+^ ATPase (PDB ID: 2DQS), a structural analog of PfATP4 was used as template. The best structural model with the most negative DOPE score was selected and visualized with PyMOL Molecular Graphics System, Version 1.7.4.5 Schrödinger, LLC^68^. The structure was further refined by using ModRefiner which is an algorithm used for high-resolution protein structure refinement at atomic-level^69^. Refined structural model, thus generated, showed the RMSD and TM-score values up to 0.567Å and 0.9975, respectively and subsequently used for docking studies. Quality validation of the resultant model as done by using online available server RAMPAGE, showed good quality with 92.1% of residues lying in the most favorable region in the Ramachandran plot.

#### Molecular docking of scaffolds 6 & 7 and PfATP4

Chemical structures of scaffold **6** and **7** were drawn using ChemDraw Ultra 12.0.2, followed by generation of their energy-minimized 3D-models by using Chem3D Pro 12.0, as described by Yadav, P. *et al*.^70^. Molecular docking studies were performed by using Autodock Vina Tools 1.5.6 to rationalize the inhibitory activities of scaffolds **6** and **7** against PfATP4^70,71^. As per the predicted ligand-binding pocket by SiteHound-web, based on total interaction energy between a chemical probe and PfATP4 structure, we ensured that the active site residues were covered while constructing the virtual grid for docking^72^. Incorporating these predicted residues, a virtual 3D grid of 24 × 28 × 24 with x, y, z coordinates of the center of energy, 38.596, −12.341 and 57.353 respectively was constructed through Autogrid module of AutoDock Tools. Top scoring docked conformations of the scaffolds were selected based on their most negative free binding energies and visualized for polar contacts (if any) with the amino acid residues of PfATP4 by using PyMOL Molecular Graphics System.

#### Immunostaining with autophagy marker, autophagy related protein, ATG8

Autophagy related protein ATG8 is a known marker for process of autophagy. During autophagy, PfATG8 co-localizes with late endosomal marker PfRAB7^41–43^. To investigate effect of scaffold **7** on this co-localization, *P*. *falciparum* cultures containing schizonts treated with scaffold **7** and solvent alone were smeared on glass slides, dried, fixed with methanol and probed with anti-PfATG8 rabbit sera and anti-PfRAB7 rat sera diluted 1:50 and 1:100, as described previously by Agarwal, S. *et al*. and Iyer, G. R. *et al*.^73,74^. After 1-hour incubation, slides were washed three times with PBS and incubated for 1 hour with Alexa 594-conjugated goat anti-rabbit IgG antibodies diluted 1:500 or Alexa-Fluor 488-conjugated goat anti-rat IgG antibodies diluted 1:200 and mounted with DAPI Antifade reagent (Molecular Devices, USA) for nuclear staining. Images were acquired in Olympus FLUOVIEW FV3000 confocal microscope using Olympus cellSens dimensions imaging software. Colocalization analysis from both channels (red: PfATG8; green: PfRAB7) was performed on a pixel by pixel basis using the same software. Every pixel in the image was plotted in the scatter diagram based on its intensity level from the channels, followed by estimation of Pearson’s correlation coefficient (r). Three-dimensional views of the images were acquired by Imaris - microscopy image analysis software. For the estimation of ‘r’, an infected and stained RBC was selected using the Region of Interest (ROI) tool provided in the software. From this ROI area, the ‘r’ value was noted as given by the software in ROI statistics^61^.

#### Mitochondrial membrane potential measurement via JC-1 staining

The fluorescence of the MitoProbe™ JC-1 dye (Molecular Probes, Life Technologies) is dependent on the mitochondrial membrane potential state. Whenever there is loss of mitochondrial membrane potential (ΔΨm), the dye remains in its monomeric form (emission maximum at 530 nm, green fluorescence), but normal or higher membrane potential results in formation of JC-1 aggregates (emission maximum at 590 nm, red fluorescence)^45^. Protocol was followed as described previously^61^. Briefly, parasites after treatment with potent scaffold **7** were harvested and incubated with 2 μM JC-1 for 30 minutes at 37 °C, washed twice with PBS and imaged immediately using Olympus FLUOVIEW FV3000 confocal microscope. For each condition, the ratio of red to green fluorescence in 100 parasites from randomly selected fields was estimated. For the estimation of fluorescence intensity in each channel, an infected and stained RBC was selected using the Region of Interest (ROI) tool provided in the Olympus cellSens dimensions imaging software. From this ROI area, the fluorescence intensity was noted as given by the software in ROI statistics. These values were used to calculate the ratio of red to green fluorescence in 100 RBCs in those fields for each set of parasite culture.

To conclude if scaffold **7** does not interfere with JC-1 aggregation in a cell free system, fluorescence emission spectroscopy was done in the absence and presence of scaffold **7**. The protocol was followed as described by Perelman, A. *et al*.^75^. Briefly, JC-1 was dissolved to a final concentration of 2 μM in the presence of <1% (0.065%) or 35% DMSO. Emission spectra at 488 nm were determined by Varioskan Flash multimode reader (Thermo Scientific).

#### TUNEL mediated DNA fragmentation assessment

DNA fragmentation in treated and untreated parasites was evaluated by using APO-BrdU™ TUNEL Assay Kit (Molecular Probes, Invitrogen) and analyzed by flow cytometry, as previously described^61^. In brief, samples were fixed with 4% paraformaldehyde (Sigma-Aldrich, Fluka Chemicals) and 0.0075% glutaraldehyde in PBS for 30 minutes at RT, washed with PBS and permeabilized by 0.01% Triton- 100. Following this, RBCs were incubated with a DNA labeling mix of TdT enzyme, BrdUTP and dH_2_O for 2 hours at 37 °C in a temperature controlled water bath. At the end of incubation period, labelled parasite was washed with rinse buffer provided in the kit at 2000 rpm for 5 minutes. To analyze the apoptotic cells, parasites were stained with Fluor^R^ 488 dye–labeled anti-BrdU antibody at 1:100 dilutions for 30 minutes at RT. Samples were then analyzed by flow cytometry and confocal microscopy.

## Supplementary information

Supplementary Information
