# Supplementary material for: Natural Product Inspired Novel Indole based Chiral Scaffold Kills Human Malaria Parasites via Ionic Imbalance Mediated Cell Death
Source: Sci Rep. 2019 Nov 28;9:17785. doi: 10.1038/s41598-019-54339-z (PMC6882913; doi:10.1038/s41598-019-54339-z)
Supplement: Supplementary file 1 — Supplementary Information [file 41598_2019_54339_MOESM1_ESM.pdf]

Natural Product Inspired Novel Indole based Chiral Scaffold Kills Human Malaria Parasites via Ionic Imbalance Mediated Cell Death

Poonam Dangi[1#], Ravi Jain[1#], Rajanikanth Mamidala[2], Vijeta Sharma[1], Shalini Agarwal[3], Chandramohan Bathula[4], M. Thirumalachary[2], Subhabrata Sen[4] & Shailja Singh[1,5*]

[1]Department of Life Science, Shiv Nadar University, Gautam Buddha Nagar, 201314, India,

[2]Jawaharlal Technological University, Kukatpally, 500072, Hyderabad, India.

[3]International Centre for Genetic Engineering and Biotechnology, New Delhi, 110067, India.

[4]Department of Chemistry, Shiv Nadar University, Gautam Buddha Nagar, 201314, India.

[5]Special Centre for Molecular Medicine, Jawaharlal Nehru University, New Delhi, 110067, India.

Keywords: Natural product, indole based anti-malarial, Ion homeostasis, PfATP4, Autophagy, Apoptosis.

Running Title: Natural indole based scaffold induces apoptosis via ionic imbalance in malaria parasite.

*Correspondence should be addressed to
shailjasingh@mail.jnu.ac.in

[#] Equal contribution

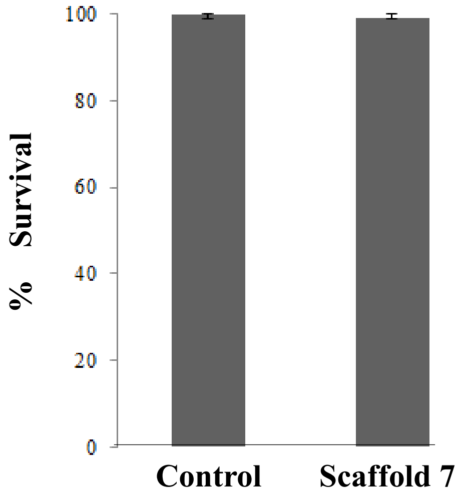

Supplementary figure 1: Scaffold **7** was tested for its cytotoxic effect on mammalian cell line, HepG2. Cells were treated with scaffold **7** at 50µM concentration (nearly double the IC_{50} value) for 24 hours. Cytotoxic effect was assessed using MTT assay. Treated HepG2 cells were as healthy as control cells.

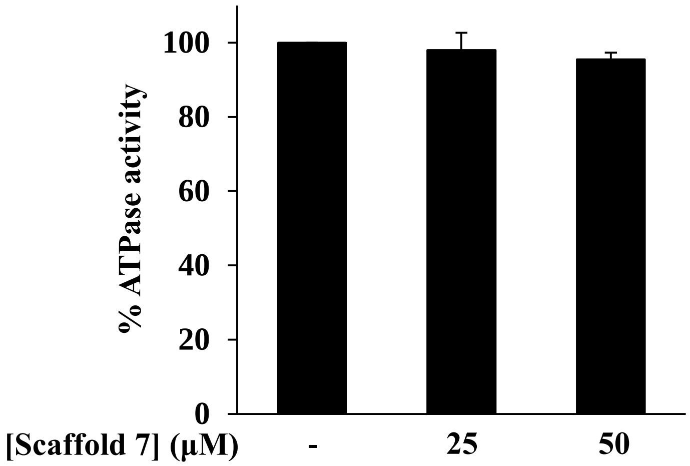

Supplementary figure 2: ATPase activity in membrane fraction of the parasite in the low (0.5 mM) [Na^+] solution, in the absence and presence of scaffold **7**. No significant change in the ATPase activity was observed after treatment with scaffold **7** in low [Na^+] solution.
